# End-to-end prognostication in pancreatic cancer by multimodal deep learning: a retrospective, multicenter study

**DOI:** 10.1007/s00330-025-11694-y

**Published:** 2025-05-23

**Authors:** Megan Schuurmans, Anindo Saha, Natália Alves, Pierpaolo Vendittelli, Derya Yakar, Sergio Sabroso-Lasa, Nannan Xue, Núria Malats, Henkjan Huisman, John Hermans, Geert Litjens

**Affiliations:** 1 Diagnostic Image Analysis Group, Radboud University Medical Center, Nijmegen, The Netherlands; 2 Department of Medical Imaging, University Medical Center Groningen, Groningen, The Netherlands; 3 Genetic and Molecular Epidemiology Group, Spanish National Cancer Research Center, Madrid, Spain; 4 Department of Medical Imaging, Radboud University Medical Center, Nijmegen, The Netherlands

**Keywords:** Pancreas, Pancreatic ductal carcinoma, Radiology, Artificial intelligence, Prognosis

## Abstract

**Objectives:**

Pancreatic cancer treatment plans involving surgery and/or chemotherapy are highly dependent on disease stage. However, current staging systems are ineffective and poorly correlated with survival outcomes. We investigate how artificial intelligence (AI) can enhance prognostic accuracy in pancreatic cancer by integrating multiple data sources.

**Materials and methods:**

Patients with histopathology and/or radiology/follow-up confirmed pancreatic ductal adenocarcinoma (PDAC) from a Dutch center (2004–2023) were included in the development cohort. Two additional PDAC cohorts from a Dutch and Spanish center were used for external validation. Prognostic models including clinical variables, contrast-enhanced CT images, and a combination of both were developed to predict high-risk short-term survival. All models were trained using five-fold cross-validation and assessed by the area under the time-dependent receiver operating characteristic curve (AUC).

**Results:**

The models were developed on 401 patients (203 females, 198 males, median survival (OS) = 347 days, IQR: 171–585), with 98 (24.4%) short-term survivors (OS < 230 days) and 303 (75.6%) long-term survivors. The external validation cohorts included 361 patients (165 females, 138 males, median OS = 404 days, IQR: 173–736), with 110 (30.5%) short-term survivors and 251 (69.5%) longer survivors. The best AUC for predicting short vs. long-term survival was achieved with the multi-modal model (AUC = 0.637 (95% CI: 0.500–0.774)) in the internal validation set. External validation showed AUCs of 0.571 (95% CI: 0.453–0.689) and 0.675 (95% CI: 0.593–0.757).

**Conclusion:**

Multimodal AI can predict long vs. short-term survival in PDAC patients, showing potential as a prognostic tool in clinical decision-making.

**Key Points:**

***Question*** *Prognostic tools for pancreatic ductal adenocarcinoma (PDAC) remain limited, with TNM staging offering suboptimal accuracy in predicting patient survival outcomes.*

***Findings*** *The multimodal AI model demonstrated improved prognostic performance over TNM and unimodal models for predicting short- and long-term survival in PDAC patients.*

***Clinical relevance*** *Multimodal AI provides enhanced prognostic accuracy compared to current staging systems, potentially improving clinical decision-making and personalized management strategies for PDAC patients.*

**Graphical Abstract:**

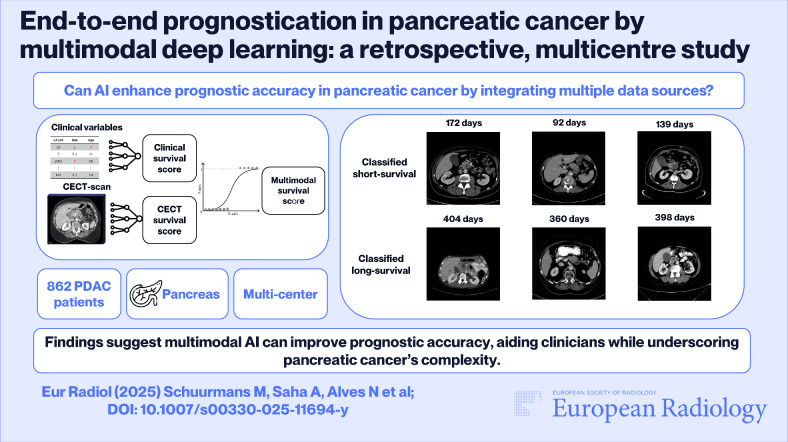

## Introduction

With a mere 5% five-year survival rate, pancreatic ductal adenocarcinoma (PDAC) remains one of the deadliest malignancies worldwide, presenting a considerable challenge to patients, clinicians, and researchers alike [1]. Imaging is crucial in stratifying PDAC patients for stage-appropriate therapies, with contrast-enhanced CT (CECT) as the major imaging modality [2, 3]. The clinical standard for cancer staging is the TNM system, which considers the extent of the tumor (T), the spread into regional lymph nodes (N), and the spread to distant sites (metastasis, M). This staging system seeks to identify distinct survival subgroups within the patient population (stages IA, IB, IIA, IIB, III, IV) and currently guides PDAC treatment trajectories. Only 20% of patients (stages I-III) are eligible for curative resection, making resection and adjuvant/neo-adjuvant therapy their primary hope for long-term survival or cure [4, 5]. Over 90% of PDAC patients undergoing curative resection experience regional cancer recurrence, distant recurrence, or metastasis [4]. Additionally, patients with the same TNM stage show considerably different overall survival rates. This variability is reflected in the prognostic performance of TNM staging, which can be quantified using the concordance index (c-index)—a measure of how well a model discriminates between patients with different survival outcomes [6]. A c-index of 0.5 indicates no better discrimination than chance, while a value of 1 represents perfect prognostic accuracy. Reported c-indexes for TNM staging areas low as 0.57 and 0.611 for resectable and unresectable patients, respectively [7–12]. Hence, it is evident that TNM staging lacks prognostic accuracy, fails to correlate significantly with overall survival, and proves unreliable in guiding treatment decisions [4, 13].

Given the limitations of conventional TNM staging, better PDAC stratification systems are urgently needed. Artificial intelligence (AI) is currently widely used in medical image analysis and cancer research, showing promising results in predicting patient outcomes for various types of carcinomas, such as larynx [14], breast [15], renal [16], colorectal [17], and pancreas. While clinicians typically use a variety of data sources to guide decision-making (for instance, combining radiology imaging with clinical or laboratory findings and histopathology assessment when available), the vast majority of AI research on PDAC prognostication using imaging relies on a single data type [18]. Multimodal AI algorithms can extract and combine information from multiple data sources and types to make more informed decisions, emulating the input conditions that clinicians currently use to address complex clinical questions such as cancer prognostication. [16, 19].

In this study, we first explored the predictive power of unimodal clinical variables and imaging AI models for discriminating short-term and long-term PDAC survivors at the time of diagnosis. Subsequently, a comprehensive multimodal AI model that integrates clinical biomarkers and imaging data was developed to evaluate its performance compared to unimodal AI.

## Materials and methods

### Ethics statement

Ethical approval was obtained from each contributing center. Data collection and analysis were done anonymously. This study was approved by the institutional review board (Radboud University Medical Centre, Nijmegen, The Netherlands, CMO2016-3045, protocol version 3, 21 September 2018), and informed consent from individual patients was waived due to its retrospective design.

### Patient cohorts

**Fig. 1:**
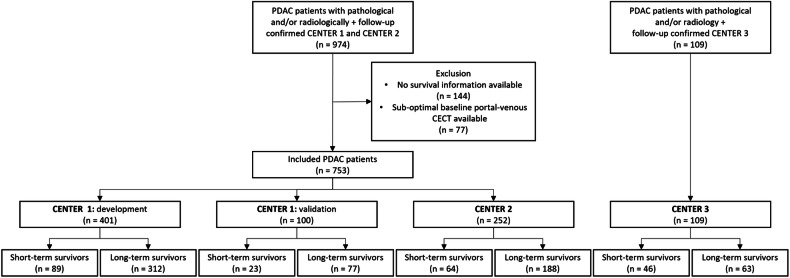
Flowchart of selected PDAC patients. There were 974 selected PDAC patients from CENTER 1 and CENTER 2. After exclusion, 753 patients remained. An additional external cohort, CENTER 3, was added, including 109 PDAC patients

This retrospective, multicenter study included anonymized CECT scans and clinical data from three medical centers (The Netherlands and Spain). Inclusion criteria were (1) baseline CECT imaging in the portal-venous phase before treatment; (2) available survival information, either the time of last contact or survival time; (3) pathology-proven PDAC or a valid document recorded by the pancreatic cancer specialized multidisciplinary advisory board, supporting the PDAC diagnosis. The exclusion criterion was suboptimal CECT imaging quality. Overall survival was followed up to death or the last date of contact. The patient inclusion diagram is shown in Fig. 1. The patient characteristics in each dataset have been summarized (Table 1).

**Table 1:** Patient characteristics for the development, internal validation, and external validation, respectively

CENTER 1 development set		CENTER 1 validation set	CENTER 2	CENTER 3
Number of patients	401	100	252	109
Age, years, median (IQR)	66.0 (58.0–73.0) (*p* = 0.207)	67.8 (59.0–72.0) (*p* = 0.060)	68.0 (59.8–73.0) (*p* = 0.261)	69.0 (62.0–78.0) (*p* = 0.310)
Female, *n* (%)	203 (50.6) (*p* = 0.498)	43 (43.0) (*p* = 0.791)	124 (49.2) (*p* = 0.213)	41 (37.6) (*p* = 0.291)
Body mass index, kg/m^2^, median (IQR)	24.7 (21.5–27.8) **(*p*** = **0.031)**	23.9 (22.1–26.8) (*p* = 0.311)	23.4 (21.2–27.5) (*p* = 0.678)	24.0 (22.3–28.0) (*p* = 0.161)
ECOG, *n* (%)	(*p* = 0.357)	(*p* = 0.059)	(*p* = 0.678)	(*p* = 0.096)
0	59 (14.7)	19 (19.0)	17 (6.7)	8 (7.3)
1	51 (12.7)	13 (13.0)	6 (2.4)	18 (16.5)
2	11 (2.7)	1 (1.0)	2 (0.8)	5 (4.6)
3	2 (0.5)	0 (0.0)	0 (0.0)	2 (1.8)
Unknown	278 (69.3)	67 (67.0)	227 (90.1)	76 (69.7)
Weight loss, kg, median (IQR)	5.0 (3.0–9.0) (*p* = 0.052)	4.5 (0.8–6.8) (*p* = 0.119)	8.0 (5.0–12.0) (*p* = 0.764)	5.0 (2.5–11.5) (*p* = 0.076)
Stage*, *n* (%)				
IA	16 (4.0)	2 (2.0)	4 (1.6)	5 (4.6)**
IB	37 (9.2)	11 (11.0)	15 (6.0)	
IIA	23 (5.7)	9 (9.0)	10 (4.0)	27 (24.8)**
IIB	97 (24.2)	24 (24.0)	55 (21.8)	
III	118 (29.4)	22 (22.0)	122 (48.4)	28 (25.7)
IV	100 (24.9)	30 (30.0)	44 (17.5)	34 (31.2)
Unknown	10 (2.5)	2 (2.0)	2 (0.8)	15 (13.8)
Resected patients, *n* (%)	189 (47.1) **(*p*** < **0.001)**	45 (45.0) **(*p*** = **0.010)**	146 (57.9) **(*p*** < **0.001)**	33 (30.3) **(*p*** < **0.001)**
Chemotherapy patients, *n* (%)	**(*p*** < **0.001)**	**(*p*** = **0.034)**	**(*p*** < **0.001)**	**(*p*** < **0.001)**
Neo-adjuvant	16 (4.0)	4 (4.0)	8 (3.8)	24 (22.0)***
Palliative/adjuvant	135 (33.7)	26 (26.0)	79 (31.3)	
Neo- and adjuvant	23 (5.7)	7 (7.0)	9 (3.6)	85 (78.0)
No	227 (56.6)	63 (63.0)	156 (61.9)	
CA19-9, U/mL, median (IQR)	237.0 (52.3–1297.8) (*p* = 0.054)	272.0 (55.0–1072.5) (*p* = 0.066)	227.0 (50.8–1554.3) **(*p*** < **0.001)**	418.4 (96.0–2089.0) **(*p*** < **0.001)**
CEA, ng/mL, median (IQR)	4.8 (3.0–8.5) (*p* = 0.509)	4.0 (2.3–6.6) **(*p*** = **0.016)**	3.0 (1.8–6.9) **(*p*** = **0.034)**	4.6 (2.6–12.8) **(*p*** = **0.046)**
Tumor size, mm, median (IQR)	31.0 (24.0–40.0) **(*p*** < **0.001)**	28.0 (22.0–38.0) **(*p*** = **0.014)**	30.0 (25.0–38.5) **(*p*** < **0.001)**	30.0 (22.5–40.0) (*p* = 0.157)
Tumor location, *n* (%)	**(*p*** = **0.036)**	(*p* = 0.112)	(*p* = 0.225)	(*p* = 0.129)
Head	276 (68.8)	72 (72.0)	172 (68.3)	75 (68.8)
Body	57 (14.2)	14 (14.0)	35 (13.9)	11 (10.1)
Tail	43 (10.7)	8 (8.0)	25 (9.9)	21 (19.3)
Unknown	25 (6.2)	6 (6.0)	20 (7.9)	2 (1.8)
Resection margin, *n* (%)	(*p* = 0.354)	(*p* = 0.231)	(*p* = 0.216)	(*p* = 0.436)
R0	65 (34.4)	20 (44.4)	65 (45.8)	16 (48.5)
R1	117 (61.9)	21 (46.7)	75 (52.8)	16 (48.5)****
R2	0 (0.0)	0 (0.0)	2 (1.4)	
Unknown	7 (3.7)	4 (8.9)	4 (2.7)	1 (3.0)
Censored survival, *n* (%)	140 (34.7)	28 (28.0)	46 (18.3)	6 (5.5)
Survivors classified, *n* (%)	Short (< 230 days): 98 (24.4) Long (≥ 230 days): 303 (75.6)	Short (< 230 days): 23 (23.0) Long (≥ 230 days): 77 (77.0)	Short (< 230 days): 64 (25.4) Long (≥ 230 days): 188 (74.6)	Short (< 230 days): 46 (42.2) Long (≥ 230 days): 63 (57.8)
Overall survival, days, median (IQR)	344 (169.0–577.0)	406 (144.8–703.8)	415 (205.8–758.5)	327 (117.0–624.0)

*n.r.* not reported

* Staged by a radiologist according to the 8th TNM edition

** Stage IA/IB and stage IIA/IIB were provided as stage I and stage II, respectively

*** Chemotherapy use was provided as a binary value: yes/no

**** Resection margin was provided as a binary value: R1/R2 vs. R0

The bold *p*-values show if there is a statistical significant difference between the short- and long survival group with respect to the variable

The model development cohort included 401 patients with all stages of PDAC acquired between 2004 and 2021 from Center 1, the Radboud University Medical Center (RUMC; Nijmegen, The Netherlands). The internal validation cohort included an additional 100 patients from CENTER 1 who were not used for model development. Two separate external validation cohorts were used. The first external validation cohort included 252 patients acquitted between 2009 and 2023 from Center 2, the University Medical Center Groningen (UMCG; Groningen, The Netherlands). The second external validation cohort included 109 patients between 2009 and 2014 from Center 3, the Centro Nacional de Investigaciones Oncológicas (CNIO; Madrid, Spain).

### Experimental design and statistics

All developed AI models predicted patient-level likelihood scores for short-term survival. Patients were stratified into the short-term survivor group or the long-term survivor group by using the median survival time of the non-resected PDAC patients in the development cohort as the threshold. The area under the receiving operating curve (AUROC), a commonly used metric for binary classification, was used to assess the model’s ability to distinguish between short-term and long-term survival. The confidence interval (CI) with 95% confidence for AUC represents the range in which the true AUC value is likely to fall, indicating the uncertainty around the model’s performance estimate. The prognostic value of the current TNM staging system was assessed using the log-rank test to compare survival between patients in different TNM stages. Statistical analysis was performed using Kaplan–Meier analysis, log-rank test, and Cox-proportional hazard test to compare survival between the groups implemented with the Lifelines packages in Python. The Scikit-learn package was used to calculate the AUROCs and 95% confidence intervals.

### Clinical model

The collected clinical variables included age, sex, carbohydrate antigen (CA) 19-9 level, carcinoembryonic antigen (CEA) level, Eastern Cooperative Oncology Group (ECOG) status, body mass index (BMI), unintended weight loss, and the 8th edition of the American Joint Committee on Cancer (AJCC) TNM stage. Expert abdominal radiologists from CENTER 1 assessed tumor location and size (Table 1). Ultimately, we used seven parameters for the clinical models: age at diagnosis, CA19-9 level, CEA level, BMI, unexpected weight loss, and ECOG performance status. All variables, except for CA19-9 and CEA levels, were normalized to the maximum and minimum values within the development cohort. The CA19-9 and CEA levels were clipped to the 95% percentile of the maximum value within the development cohort to account for extreme elevations and normalized to the maximum and minimum values within the clipped cohort. The clinical model consisted of a two-layer neural network with a binary cross-entropy loss function trained for 200 epochs using five-fold cross-validation. For each fold, missing clinical values in the training and validation sets were imputed by taking the median of the clinical variable in the training set only. The missing variables in the internal validation dataset of CENTER 1, CENTER 2, and CENTER 3 were imputed with the median of the clinical variables in the complete development cohort of CENTER 1.

### Imaging model

All CECTs were automatically segmented with a previously validated and open-source AI algorithm for pancreas parenchyma, pancreatic duct, and pancreatic tumor [20]. Segmentations from CENTER 1, CENTER 2, and CENTER 3 cohorts were manually verified and corrected by trained medical students using ITK-SNAP version 3.8.0, a specialized medical imaging software for interactive segmentation, supervised by an abdominal radiologist with 23 years of experience in pancreatic radiology. The pancreas parenchyma and pancreatic tumor segmentation are used to mask the CECT. As CECTs can be captured at different resolutions, meaning the size of individual 3D voxels can vary, all CECT images in this study were subsequently resized to the same dimensions (96, 192, 300) and uniform voxel sizes (1, 1, 1). Intensities (i.e., Hounsfield units) were clipped between [−1000, 300] to account for extremes and normalized to the maximum and minimum intensity values on the patient level. The intensity clipping range of [−1000, 300] HU was selected based on established domain knowledge as values below −1000 HU correspond to non-physiological noise or air artifacts, while values above 300 HU generally represent bone, calcifications, or contrast material, which are not relevant to our soft tissue analysis and could introduce noise into the learning process.

The imaging AI model consists of an EfficientNet-B4, implemented in MONAI (PyTorch-based), trained for 500 epochs using five-fold cross-validation [21]. The multimodal AI models combine both by adding a logistic regression using the risk scores from the unimodal AI models as input. The AI models are publicly available on GitHub (https://github.com/meganschuurmans/mosp).

## Results

**Fig. 2:**
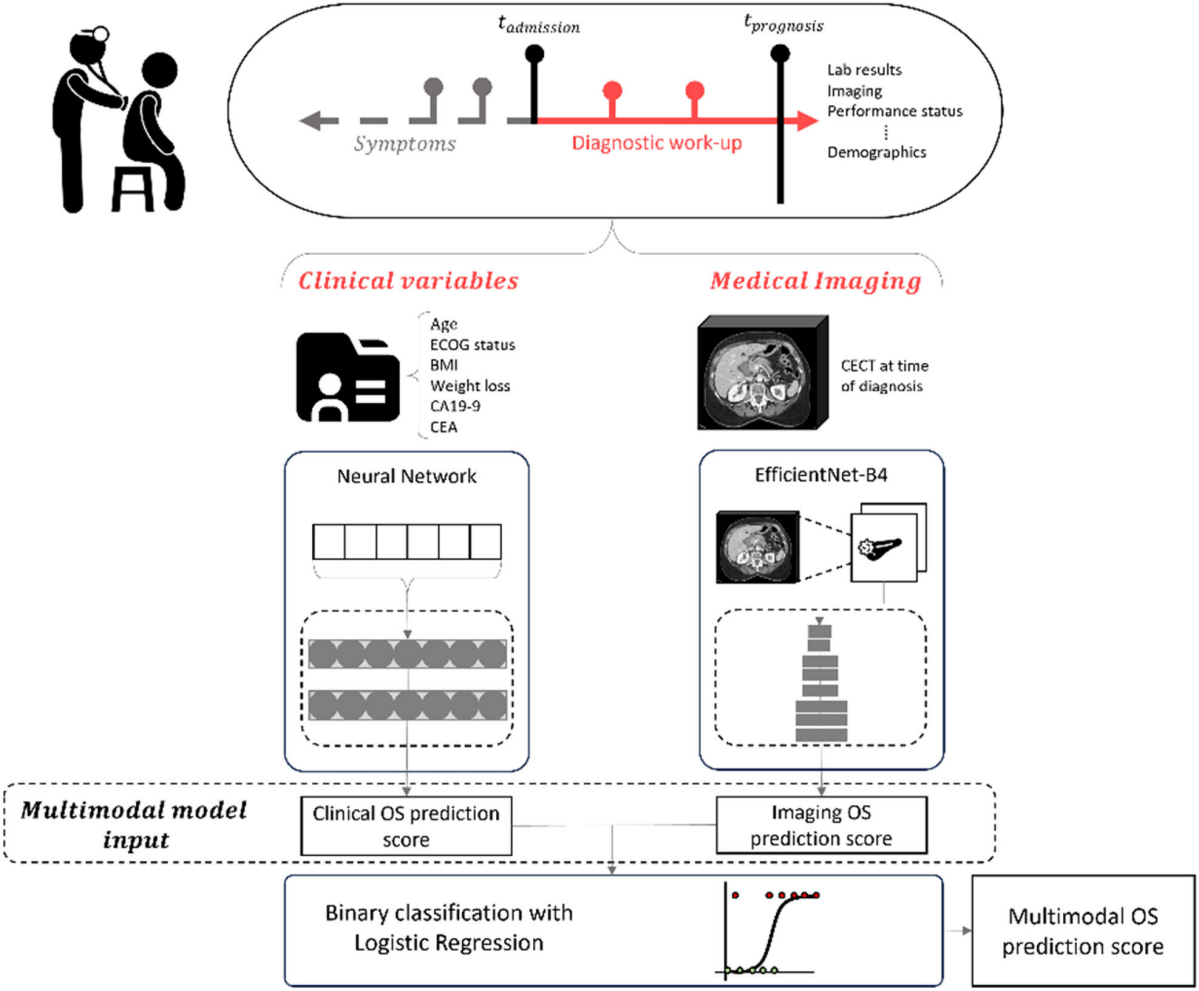
Schematic overview of the multimodal survival prediction pipeline. Overall survival (OS) scores float between 0 and 1, indicating the likelihood of a patient being a short-term survivor

**Fig. 3:**
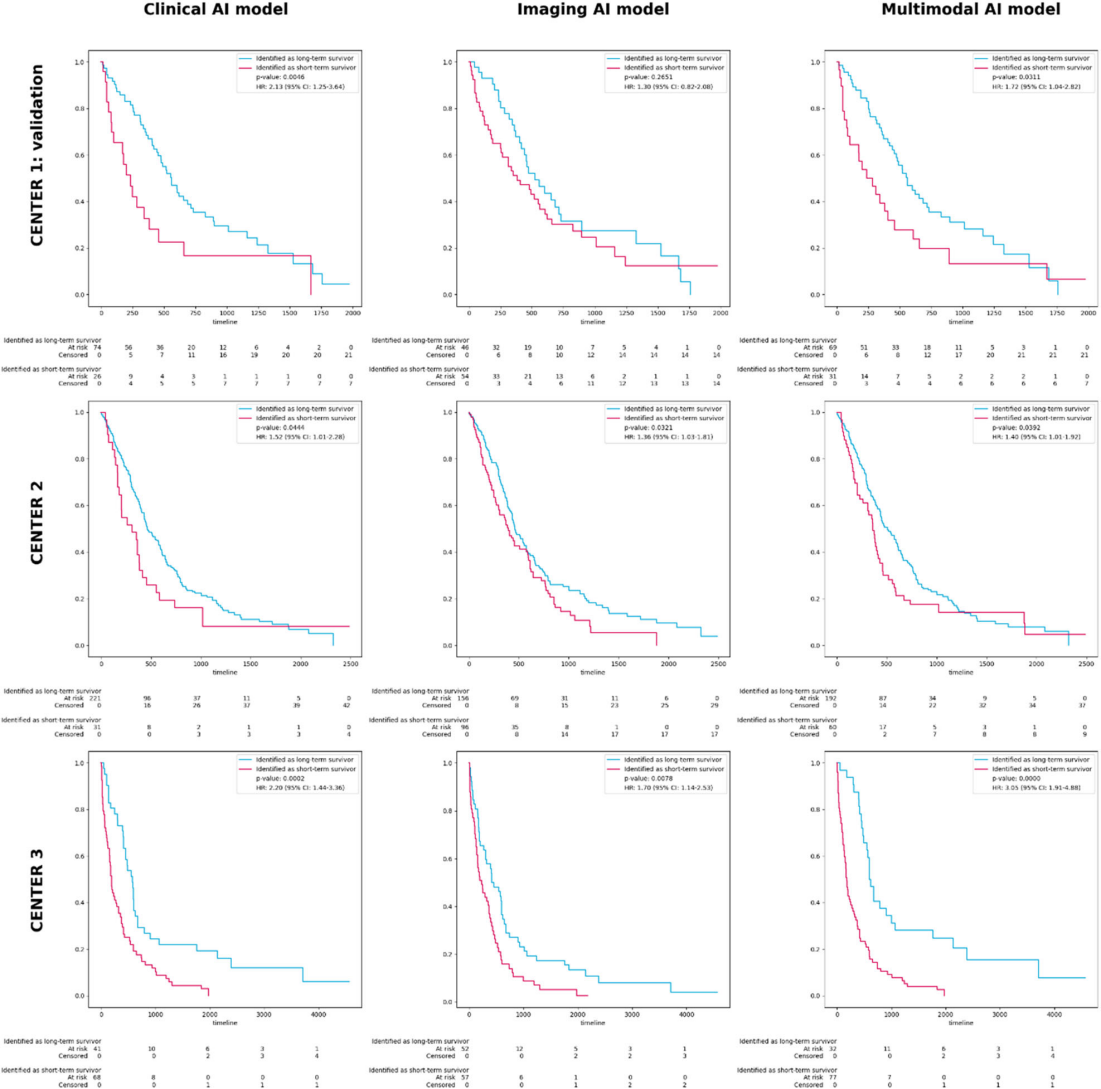
Kaplan–Meier curves for identified short (red) and long (blue) survivors for the unimodal clinical, imaging, and multimodal AI models for CENTER 1: validation cohort, CENTER 2, and CENTER 3

We developed end-to-end unimodal deep-learning models for clinical and imaging data, and a combined multimodal model (Fig. 2). The mean AUROCs of the unimodal AI models using clinical variables and imaging using five-fold cross-validation in the development cohort were 0.578 (95% CI: 0.312–0.845) and 0.575 (95% CI: 0.478–0.673), respectively. The mean AUCROC of the multimodal AI models was 0.637 (95% CI: 0.500–0.774). The AUROC for the unimodal AI models using clinical and imaging variables in the external validation sets were 0.554 (95% CI: 0.455–0.653), 0.536 (95% CI: 0.452–0.620) for CENTER 2 and 0.638 (95% CI: 0.430–0.846), 0.550 (95% CI: 0.485–0.615) for CENTER 3, respectively. The AUROC for the multimodal AI model was 0.571 (95% CI: 0.453–0.689) for CENTER 2 and 0.675 (95% CI: 0.593–0.757) for CENTER 3. In the Kaplan–Meier analysis, the deep-learning risk score demonstrated generally more significant risk stratification with the multimodal AI models (Fig. 3).

**Fig. 4:**
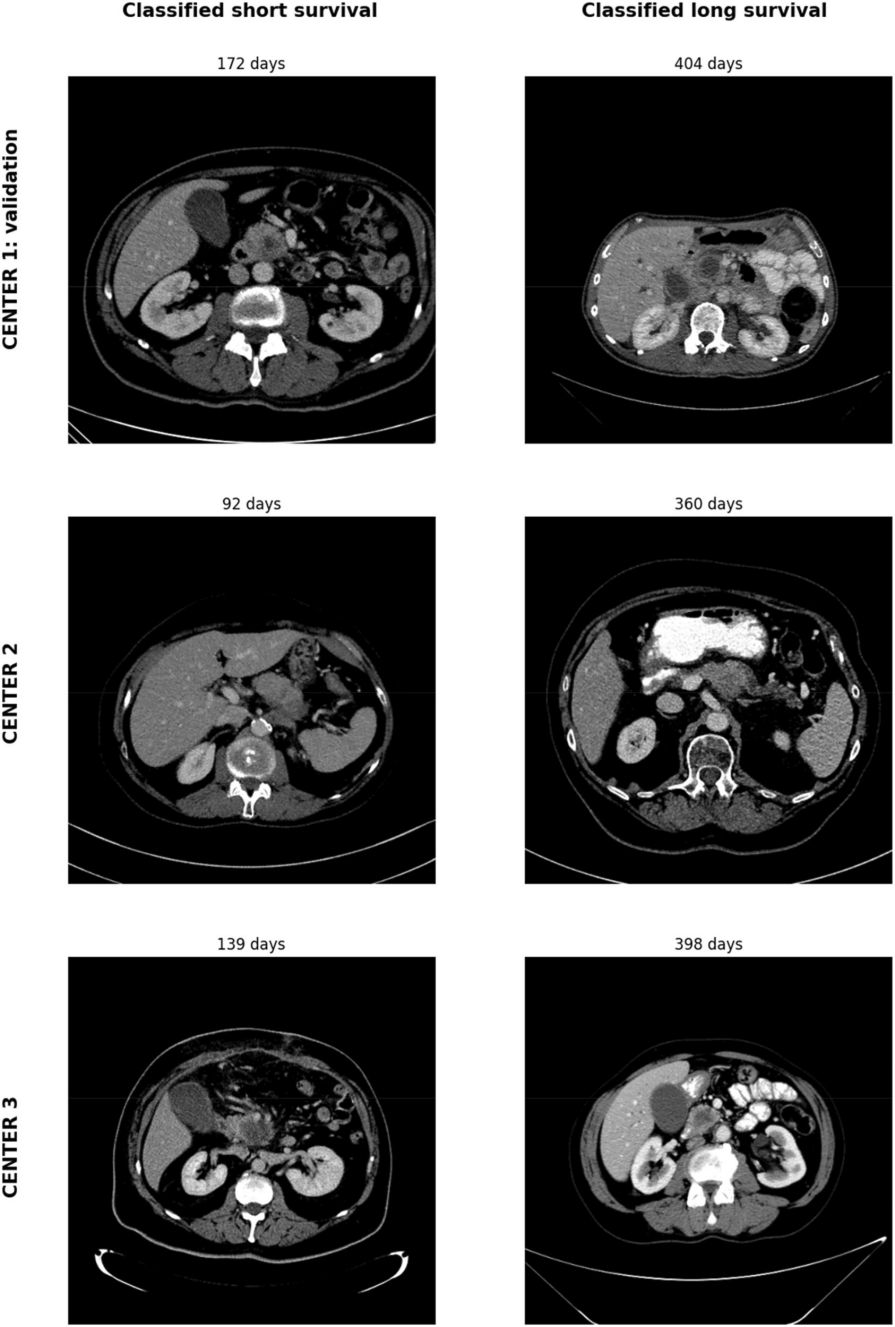
Patient examples of the identified short- and long survivors in the CENTER 1: validation set, CENTER 2, and CENTER 3

Patient examples of the output of the multimodal AI algorithms are shown in Fig. 4. The short- and long-term classified survivors from CENTER 1: validation cohort are 60-year-old and 63-year-old males, respectively. Both patients did not receive resection, presented metastasis at the time of diagnosis, and were staged with AJCC TNM score IV. The short- and long-term classified survivors from CENTER 2 are 66-year-old and 71-year-old females, respectively. Neither patient received resection. The short-term classified patient presented metastasis at the time of diagnosis, while the long-term classified patient did not. The short- and long-term classified patients were staged with AJCC TNM score IV and III, respectively. The short- and long-term classified survivors from CENTER 3 are 74-year-old male and 72-year-old female, respectively. Neither patient received resection. The short- and long-term classified patients were staged with AJCC TNM score III and IV, respectively.

**Fig. 5:**
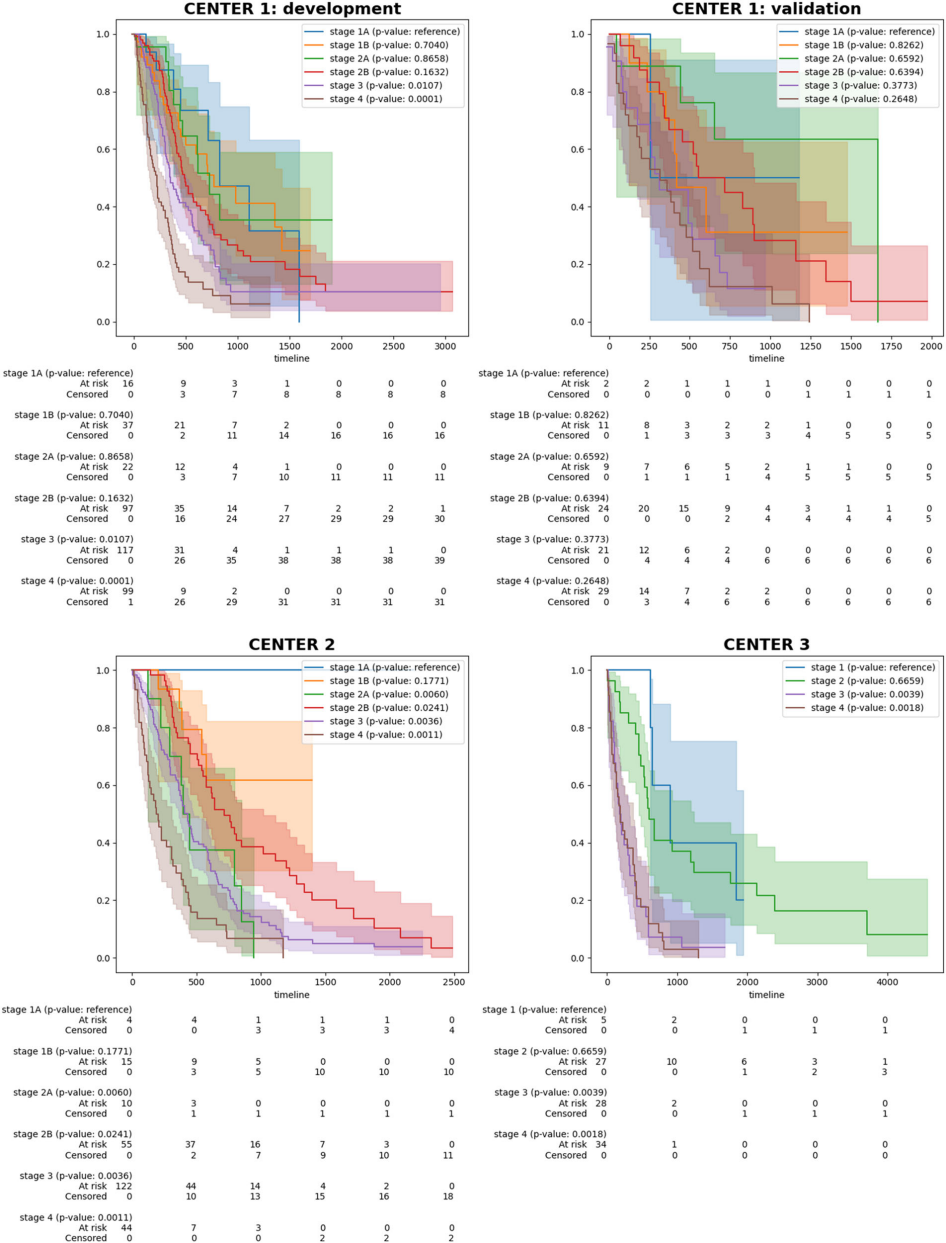
Kaplan–Meier curves for all identified TNM stages in the CENTER 1: development cohort, CENTER 1: validation cohort, CENTER 2, and CENTER 3

Additionally, we have plotted the Kaplan–Meier curves for the TNM stages in the development and validation cohorts, using the log-rank test to compare stage IA with stages IB-IV (Fig. 5). This analysis provides insight into the discriminative ability of TNM staging, as reflected in the statistical significance of survival differences across all stages. Notably, data from CENTER 3 lacked stage IB and IIB classifications. The *p*-values exhibit substantial heterogeneity across different centers. For the development cohort at CENTER 1, the *p*-values for stages IB-IV are 0.7040, 0.8658, 0.1632, 0.0107, and 0.0001, respectively, with the most statistically significant difference observed between stage I and stage IV. In the validation cohort of CENTER 1, *p*-values for stages IB-IV are 0.8262, 0.6592, 0.6394, 0.3773, and 0.2648, respectively, indicating no significant differences when compared to stage I. At CENTER 2, *p*-values for stages IB-IV are 0.1771, 0.0060, 0.0241, 0.0036, and 0.0011, respectively, suggesting higher statistical significance than CENTER 1, although the Kaplan–Meier curves for stages 2A and 3 are intertwined. Lastly, at CENTER 3, the *p*-values for stages II-IV are 0.6659, 0.0039, and 0.0018, respectively, showing significant differences between stages I and IV, but low significance between stages III and IV, as shown by the overlapping curves.

## Discussion

In this study, we developed a deep learning-based multimodal prognostic-stratification system for PDAC patients. Our open-source multimodal models showed promising predictive performance in internal validation cohorts and two external, international cohorts.

PDAC has a low prevalence but lacks typical early-stage symptoms and is highly malignant. Current TNM staging systems guide treatment plans and impact survival time significantly. Several studies have evaluated the TNM staging performance for predicting overall survival in PDAC patients, showcasing its shortcomings in stratifying patient outcomes. The most extensive cohort study, involving 125,183 patients from the USA and Europe, reported limited survival improvements between stages, with primarily statistically insignificant differences between stages I-II and III-IV [22]. Furthermore, the study lacked a detailed analysis of survival outcomes between individual stages, which is crucial for refining treatment strategies and prognostication. Another recent study assessed the latest TNM edition for prognostic accuracy in PDAC patients, utilizing a large cohort of 1043 patients. The findings showed no significant survival difference between stages IB and IIA. Additionally, a majority of patients experienced false-negative lymph node staging. These results underscore that the current TNM staging system is not well-suited for clinical staging of PDAC [13]. The performance of the latest TNM edition for prognostic accuracy was also validated in a nationwide study in the Netherlands, including 750 resected patients, confirming it still lacks prognostic value (c-index: 0.59) [12]. Despite these limitations of TNM staging, there is a notable lack of AI models capable of accurately stratifying PDAC patient survival at the time of diagnosis [18]. This gap emphasizes the need for innovative approaches to improve early prognostic assessment in PDAC.

Currently, there are no clinically accepted thresholds to differentiate short- from long-term PDAC survivors. Past research has explored various thresholds spanning from 3 months to 5 years [23–25]. Yet, none of these thresholds have been conclusively established as clinically significant or definitive in predicting survival times. In this study, we used the median survival time of patients who underwent non-curative treatment (230 days) as a threshold. In the context of the application of this AI model, this serves as a relevant time point as it helps avoid treating patients who are not predicted to survive, even up to the survival time of those receiving no curative treatment.

Research on survival outcomes in PDAC is limited, with relatively few studies published in this field. Existing studies are often constrained by small sample sizes, whereas our study, comprising 862 cases, represents the largest cohort analyzed to date. Additionally, prior investigations frequently focus on a single stage of disease, limiting their generalizability; in contrast, our dataset provides a more comprehensive representation across all stages of PDAC [23]. Furthermore, our study is the only multimodal investigation to date, integrating diverse data types for improved prognostic accuracy.

Recent high-impact studies focus on innovative staging strategies with AI to enhance prognostic accuracy and better guide treatment decisions for different types of cancer [14–17, 23, 26]. While most AI research in PDAC has focused on unimodal approaches, recent findings suggest that integrating information from multiple data sources enhances predictive power [19, 27–30]. Our findings align with these results, underscoring the advantages of multimodal AI in the prognostic stratification of PDAC patients over current clinical TNM staging systems. Future studies incorporating other data modalities, such as histopathology and genomics, may improve the accuracy of PDAC prognostic prediction.

This study comes with limitations. First, all cases were collected retrospectively, resulting in a mix of consecutive patients and convenience samples. Second, to maximize the dataset for AI development, patients are acquired over a relatively larger time span with various acquisition protocols for collecting clinical variables, imaging, and changing staging systems. Third, patients were not stratified based on treatment, as this would result in a limited dataset, which hinders AI development and generalizability. Our results provide strong evidence that AI can improve PDAC prognostication compared to current TNM staging. However, future studies considering treatment-specific cohorts are necessary before such AI models can be applied in clinical practice.

In summary, we developed and validated deep learning-based unimodal and multimodal models that use clinical information and CECT at the time of diagnosis to directly predict survival in all stages of PDAC patients. The findings indicate that multimodal AI can improve prognostic accuracy, potentially aiding clinicians in making better-informed treatment decisions while highlighting the inherent complexity and difficulty of this task for pancreatic cancer. Our multimodal AI biomarker shows superior stratification capability compared to the currently clinically used TNM staging system, as demonstrated by Kaplan–Meier analysis, even though receiver operating characteristic analysis reveals that its prognostic value is still lower compared to other cancer types. This study represents a significant step forward in personalized medicine for pancreatic cancer management. We have open-sourced our model, making it easy for other research groups to reuse, reproduce, or extend our approach.

## Abbreviations

AI: Artificial intelligence
AUROC: Area under the receiver operating characteristic
CECT: Contrast-enhanced computed tomography
OS: Overall survival
PDAC: Pancreatic ductal adenocarcinoma`
